# *In silico* Analyses of Core Proteins and Putative Effector and Immunity Proteins for T6SS in Enterohemorrhagic *E. coli*

**DOI:** 10.3389/fcimb.2020.00195

**Published:** 2020-05-05

**Authors:** Jaime Vazquez-Lopez, Fernando Navarro-Garcia

**Affiliations:** Department of Cell Biology, Centro de Investigación y de Estudios Avanzados del IPN (CINVESTAV-IPN), Mexico City, Mexico

**Keywords:** enterohemorrhagic *E. coli*, type 6 secretion system, effector proteins, Rhs, bacterial competition, pathogenesis

## Abstract

Shiga-toxin-producing *Escherichia coli* (STEC) has become an important pathogen that can cause diarrhea, hemorrhagic colitis and hemolytic uremic syndrome (HUS) in humans. Recent reports show that the type VI secretion system (T6SS) from EHEC is required to produce infection in a murine model and its expression has been related to a higher prevalence of HUS. In this work, we use bioinformatics analyses to identify the core genes of the T6SS and compared the differences between these components among the two published genomes for EHEC O157:H7 strain EDL933. Prototype strain EDL933 was further compared with other O157:H7 genomes. Unlike other typical T6SS effectors found in *E. coli*, we identified that there are several *rhs* family genes in EHEC, which could serve as T6SS effectors. *In-silico* and PCR analyses of the differences between *rhs* genes in the two existing genomes, allowed us to determine that the most recently published genome is more reliable to study the *rhs* genes. Analyzing the putative tridimensional structure of Rhs proteins, as well as the motifs found in their C-terminal end, allowed us to predict their possible functions. A phylogenetic analysis showed that the orphan *rhs* genes are more closely related between them than the *rhs* genes belonging to *vgrG* islands and that they are divided into three clades. Analyses of the downstream region of the *rhs* genes for identifying hypothetical immunity proteins showed that every gene has an associated small ORF (129-609 nucleotides). These genes could serve as immunity proteins as they had several interaction motifs as well as structural homology with other known immunity proteins. Our findings highlight the relevance of the T6SS in EHEC as well as the possible function of the Rhs effectors of EHEC O157:H7 during pathogenesis and bacterial competition, and the identification of novel effectors for the T6SS using a structural approach.

## Introduction

Shiga-toxin-producing *Escherichia coli* (STEC) bacteria, including enterohemorrhagic *E. coli* (EHEC), are intestinal pathogens responsible for diseases such as enterohemorrhagic colitis and hemolytic uremic syndrome (HUS). HUS is a serious complication that may cause renal failure and heart damage, resulting in death in 10% of the affected (Croxen et al., 2013). Antibiotic treatment against STEC infection increases HUS risk (Wong et al., 2000), and EHEC infectious dose is under 50 CFU (Tilden Jr et al., 1996), thus placing STEC as an important health risk for epidemic diseases. Cows are the principal STEC reservoir, in which the bacteria live as a commensal and do not cause infection, along with vegetables irrigated with contaminated waters, which have caused important outbreaks (Frank et al., 2011). Nowadays EHEC serotype O157:H7 and the hybrid strain between an enteroaggregative *E. coli* and STEC (EAEC/STEC O104:H4) are the most commonly associated strains to epidemic outbreaks (Yang et al., 2017). On one hand, EHEC O157:H7 has a locus of enterocyte effacement (LEE), which encodes a type 3 secretion system (T3SS) used for injecting effectors that subvert the cell physiology, causing the characteristic attaching and effacing (A/E) lesions (Schmidt, 2010). On the other hand, EAEC/STEC O104:H4 has the aggregative adherence fimbriae like AAFI as well as multiple antibiotic resistance genes (Rohde et al., 2011; Navarro-Garcia, 2014). Even if both serotypes have a different origin and have few virulence factors in common, both have the *stx2* gene, which encodes the Shiga toxin 2. The presence of this toxin in both genomes is probably due to horizontal gene transfer from a O157:H7 strain to EAEC strain 55989, the presumptive parental strain of EAEC/STEC O104:H4 (Rohde et al., 2011).

Both EHEC EDL933, an O157:H7 serotype used as a reference strain, and EAEC/STEC O104:H4, encode a type VI secretion system (T6SS) (Journet and Cascales, 2016), a nanomachine comprised from 13 *core* elements that make up for four defined structures: a membrane complex formed by TssJLM that attaches the T6SS to the inner membrane and makes the intermembrane pore; a baseplate comprised by TssEFGHK that connects the tail to the membrane complex and helps with the T6SS disassembly once it has contracted; a tail made up of stacked Hcp hexamers with a spike formed by a VgrG trimer; and a contractile sheath comprised by TssBC, which expel the Hcp tail to the exterior of the bacterial cell, while TssA regulates the T6SS assembly and contraction (Navarro-Garcia et al., 2019; Schneider et al., 2019). The core components are grouped in a genomic island and present homology with some of the components of the T4 bacteriophage machinery (Pukatzki et al., 2007; Leiman et al., 2009). The T6SS punctures other cell membranes, which can be from eukaryotic or prokaryotic organisms, and injects effectors directly into the cytoplasm that mediate bacterial competition and pathogenesis (Ma and Mekalanos, 2010; Basler et al., 2013). Effectors are usually classified into three families: (i) phospholipases that degrade the plasma membrane; (ii) murein-hydrolases and amidases that attack the cell wall; and (iii) nucleases which target DNA or RNA. The genes encoding those effectors are usually part of a bicistronic operon that also encodes an immunity protein, which binds to the effector protein and prevents autointoxication (Bingle et al., 2008). Finally, there are other effectors with non-canonical activities, including ADP ribosylation, ion chelation or even actin crosslinking (Lien and Lai, 2017).

Wan et al. (2017) have already shown that the T6SS of EHEC strain EDL933 is functional in an isogenic mutant for the master regulatory protein H-NS, and showed that EHEC can translocate KatN, a catalase effector, into the macrophage upon phagocytosis. KatN neutralizes iROs present in the phagosome, thus avoiding bacterial killing. Wan et al. (2017) also showed that T6SS is essential to cause disease in a murine model, as isogenic mutants for *clpv* were unable to provoke death in the infected mice. ClpV is an ATPase that is essential for T6SS function by recycling the sheath components (Douzi et al., 2016). Nonetheless, mice death was independent of KatN, implying that other effectors acting as virulence factors in EHEC strain EDL933. Recently, Rhs family proteins have been associated with interbacterial competition in several STEC strains. Rhs family proteins are known for containing RHS repeats (recombination hotspot), often contain a PAAR domain that interacts with VgrG proteins (Bondage et al., 2016), and also have a C-terminal region with a nuclease, protease or deaminase activity (Ma et al., 2017b). In this work, we explore the differences between the T6SS genes and the putative effectors in two published genomes of EHEC O157:H7 strain EDL933 (Perna et al., 2001; Latif et al., 2014). Using bioinformatics analyses we could identify both the core genes of the T6SS as well as several putative effectors, and we provide evidence that the most-recently published genome (Latif et al., 2014) better represents the T6SS components and effectors. Additionally, analyzing sequence motifs and the modeled structure we identified several putative Rhs effectors, as well as the associated putative immunity proteins for each Rhs.

## Methods

### Strains Used in This Study

EHEC strain EDL933 was kindly provided by Dr. Jose Luis Puente (Riley et al., 1983; Arenas-Hernández et al., 2014). The genomes bioinformatics comparison was made from EHEC strain EDL933 ATCC 700727 in the genome of Perna et al. (2001) (GenBank accession number: AE005174.2) and the genome of Latif et al. (2014) (GenBank accession number: CP008957.1). *E. coli* strain HB101 was obtained from ATCC 33694. The strains were grown overnight in LB plates with 1.5% agar at 37°C.

### Bioinformatics Analyses

Motif searching for putative T6SS effectors was performed using the PROSITE, NCBI-CDD and Pfam databases (Marchler-Bauer et al., 2010; Sigrist et al., 2012; Finn et al., 2013) with the GenomeNet search engine. An *e-value* cutoff score of 0.01 was used. The tridimensional models were calculated using I-TASSER software (Yang and Zhang, 2015). For the analyses of the possible effector activity of the Rhs proteins, only the C-terminal end of each protein was modeled. The search for T6SS islands was performed using MacSyDB (Abby et al., 2016) and SecReT6 (Li et al., 2015). Relationship analyses between different Rhs proteins was conducted using STRING version 11 (Szklarczyk et al., 2019). Prediction of signal sequences was made using SignalIP (Juncker et al., 2003). Evolutionary analyses were conducted in MEGA X (Kumar et al., 2018) from the nucleotide sequences form the genome published by Latif et al. (2014), using the Maximum Likelihood method and Tamura-Nei model (Tamura and Nei, 1993). The tree presented is the consensus of 100 bootstrap repetitions (Felsenstein, 1985).

### Primer Designing and Amplification for *rhsA* and *rhsC* Genes in EHEC Strain EDL933 DNA

Three primers were designed using Primer BLAST (Ye et al., 2012). The forward primer was designed to aligning with the *rhsA* gene in both genomes (*z5014* and *EDL933_4854*), while a genome-specific reverse primer was designed for each published genome (Perna et al., 2001; Latif et al., 2014). The sequence of the forward primer was 5′-CGCTATCTTTACGACCCGCT-3′. The primer rhsA1R with the sequence 5′-GGCAAGGGGAATGGTCTAGG-3′ was complementary with the *rhsA* gene (*locus z5014*) according to Perna et al. (2001), whereas the primer rhsA2R with the sequence 5′-GATGTGGGGGTACCATGCC-3′ was complementary to the *rhsA* gene (locus *EDL933_4854*) according to Latif et al. (2014). For the amplification of the *rhsC* gene (*locus z0847*), the primers rhsAF and rhsCR (sequence 5′-TAGGCGGTTTGTTGGGTCTC-3′) were used. The optimal aligning temperature for all the combinations were experimentally obtained by PCR using a temperature gradient and a final primer concentration of 0.4 μM. The expected product was 800 bp for rhsA1R primer and 875 bp for rhsA2R, while a fragment of 832 bp fragment was expected for rhsCR. Chromosomal DNA from EHEC strain EDL933 was used as template. After PCR, the products were separated in a 1.5% agarose gel and then stained with ethidium bromide.

## Results and Discussion

### *In-silico* Analysis of the T6SS Island in EHEC Strain EDL933

**Figure 1:**
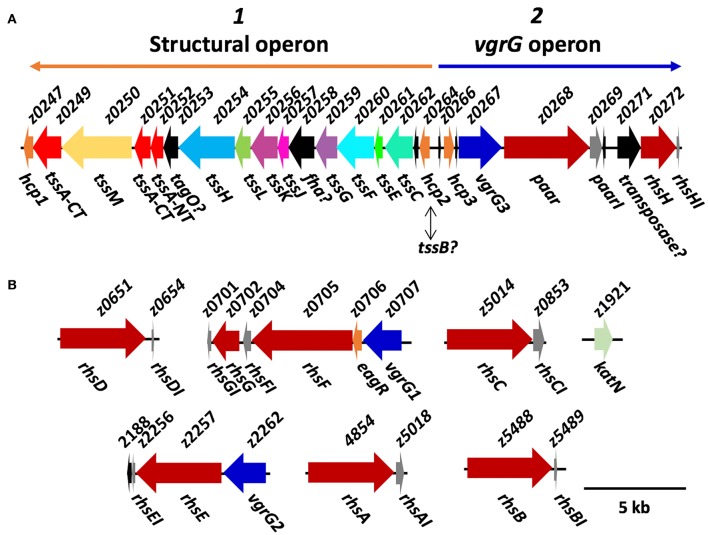
Diagram of the organization of T6SS genes in EHEC strain EDL933. **(A)** Organization of the T6SS island components, in which the genes of unknown function are shown in black. Orange and blue arrows are showing the localization of the putative operon 1 and 2 in the island (named here structural and *vgrG* operons). **(B)** Putative T6SS effectors. The *rhs* genes found in EHEC strain EDL933 are shown in red, and the *vgrG* homologs are shown in blue. KatN, the only confirmed T6SS effector to date is shown in green, while the immunity proteins are shown in gray. This diagram was constructed based on CP008957 genome published by Latif et al. (2014), and the genes only found in this genome lack the z-prefix.

Three distinct families of T6SS exist, which differ in their genetic organization as well as in the homology of their components (T6SS-1, T6SS-2 and T6SS-3) (Journet and Cascales, 2016). T6SS-1 and T6SS-3 have been associated with bacterial competition as well as with the invasive capacity of certain *E. coli* pathotypes, while T6SS-2 is commonly found in intestinal strains, including STECs like EHEC EDL933 and EAEC/STEC O104:H4 (Journet and Cascales, 2016). Wan et al. (2017) described the T6SS island in EHEC strain EDL933 using the genome published by Perna et al. (2001), which was obtained via shotgun sequencing as described by Mahillon et al. (1998). However, recent reports of structural proteins and the publication of a gapless genome without ambiguities for EHEC strain EDL933 using PacBio and Ilumina sequencing (Latif et al., 2014) allowed us to perform a deeper analysis of the genetic island (Figure 1A). We found that the island encodes the 13 core components of the T6SS (TssA, TssBC, TssEFGHK, TssJLM, Hcp, and VgrG), which appears to be divided into two general operons: the first one (named here structural operon) would be codified on the complementary DNA strand and would contain both the baseplate components (TssEFGHK) and the membrane complex components (TssJLM). Besides, this operon includes two hcp genes (*tssD*, loci *z0247*, and *z0264*) and a *tssC* gene, an essential component of the T6SS sheath. Analyzing the genomes from O157:H7 strains in GenBank, we found that 137 strains out of 145 possessed the T6SS-2 structural genes and only 8 did not (strains 09BKT002497, 121, 262, 611, EC10, EC4024, GZ-021210/cattle, and ZAP430). No significant similarity with T6SS-1 or T6SS-3 structural genes from EAEC 042, a reference strain for these T6SS families, was found in O157:H7 genomes. The second operon (named here *vgrG* operon) would be on the main DNA strand containing a group of genes that encode the tail components such as another *hcp* gene (*z0266*) and a *vgrG* gene (*tssI, z0267*). Additionally, in this operon, we found two genes downstream *vgrG*, and one of them encodes an Rhs family protein with a PAAR domain (*z0268*). Interestingly, both the *rhs* family gene and the gene directly downstream (*z0269*) were not found in every T6SS-2 islands, such as EAEC 042, EAEC 55989, or EAEC/STEC O104:H4, as previously reported (Journet and Cascales, 2016). This suggests that this pair of genes encode a T6SS effector as well as its associated immunity protein. Finally, *z0268* and *z0269* were found only in 66 EHEC O157:H7 strains from the lineage I and were absent in 79 strains from lineage II (Zhang et al., 2007).

One particularity of the T6SS island in EHEC strain EDL933 is that it contains three homologs of *hcp* genes (*z0247, z0264* and *z0266*) (Wan et al., 2017), so we decided to further analyze them. *hcp1* (*z0247*) is not recognized as an ORF in Latif et al. (2014), even though that region is 100% identical to Perna et al. (2001). This might be because there is a little overlapping between the 5' end of locus *z0247* and 3' end of locus *z0249*, so perhaps the region in this latter analysis was not properly recognized by the annotation software, RAST. For *hcp2* (*z0264*), it has already been shown that EHECΔ*hns*, an isogenic mutant for the master regulatory protein H-NS repressing the T6SS expression in several bacteria, secretes the Hcp2 protein to the supernatant (Wan et al., 2017). However, upon inspecting the motifs found in the protein, we found a PF05591 motif, which may be found both in Hcp2 and TssB. Remarkably, the island reported by Wan et al. (2017) lacks the *tssB* gene, an essential component of the T6SS (Zhang et al., 2014). Our analyses using MacsyDB and SecReT6 (Li et al., 2015; Abby et al., 2016) suggested that the gene is not *hcp* but *tssB*, but experimental data is required to completely clarify the identity of this gene. Finally, none of the three *hcp* genes belongs to the Hcp-ET family reported by Ma et al. (2017a), and no known effector domains fused with the Hcp core were found that might participate during pathogenesis or interbacterial competition in EHEC strain EDL933.

Furthermore, *tssA* gene (*z0251*) is half as long as the reported genes in other strains, and after inspecting the region between the loci *z0251* and *z0252* (Perna et al., 2001), we found that the gene seems to be interrupted, as a missing cytosine in the 249,998th nucleotide producing a stop codon. In fact, in the genome published by Latif et al. (2014), the segment containing z0251 and z0252 is recognized as a single pseudogene, even though this region is 100% identical as in the genome published by Perna et al. (2001). Being TssA an essential protein for the T6SS function, it would be odd that the gene was mutated, especially when the functionality of the T6SS has already been confirmed in this strain (Wan et al., 2017). Interestingly, Zoued et al. (2016) demonstrated that both N-terminal and C-terminal regions of TssA interact with different components of the T6SS, and it is possible that, even if the gene is divided in two, both regions could properly oligomerize and perform TssA function during T6SS biogenesis. About a third of the O157:H7 strains we analyzed showed the same single-nucleotide deletion, while the rest encoded a full length TssA protein. Additionally, *z0249* also encodes a protein of the cluster of differentiation 3515 (COG3515) that would also correspond to the C-terminal fraction of TssA, although the identity between Z0251 and Z0249 is rather low (41.67% and 7% of query cover). The TssA C-terminal region forms dodecamers, suggesting that the gene could be duplicated on the genome, but the low homology could imply that both loci perform different activities during T6SS biogenesis and/or function.

Outside of the T6SS island, we found two islands that encode VgrG proteins (*vgrG* islands, Figure 1B), which Uchida et al. (2013) renamed *vgrG1* (*z0707*) and *vgrG2* (*z2262*), while the locus *z0267* inside the T6SS island was named *vgrG3*. Interestingly, downstream of *vgrG1*, we found an hypothetical protein with a domain of unknown function 1795 (DUF1795), which has been found to interact with Rhs and VgrG proteins in *Serratia marcescens* (Alcoforado Diniz et al., 2015), and is essential for T6SS-mediated translocation of Rhs proteins in several STECs (Ma et al., 2017b). The three VgrG proteins have ≥90% amino acid identity and resemble other VgrG proteins of the T6SS-2. Interestingly, the same 8 STEC O157:H7 strains that were negative for structural genes were also negative for *vgrG* genes, while the other possessed at least one *vgrG* homolog in their genome, having up to five copies of *vgrG*.

Several T6SS effectors are VgrG proteins that have a C-terminal extension with enzymatic activity (Ma and Mekalanos, 2010; Suarez et al., 2010; Brooks et al., 2013; Sana et al., 2015). However, none of the VgrG homologs in EHEC O157:H7 strains had a known effector domain in its C-terminal, though we found that the C-terminal region was highly divergent while the N-terminal region was conserved. T6SS-translocated proteins are carried by VgrG proteins, and those effectors usually interact with the C-terminal region of VgrG (Hachani et al., 2014; Whitney et al., 2014). Thereby, we postulate that the three different VgrG proteins present in EHEC O157:H7 strains carry different effectors to the prey cell.

Wan et al. (2017) already demonstrated that T6SS in EHEC EDL933 is functional in an *hns* isogenic mutant and that it can translocate the KatN catalase upon macrophage phagocytosis, thus neutralizing the iROs in the phagosome and avoiding bacterial death. They also showed that the T6SS is essential to cause disease in a murine model, as the isogenic *clpV* mutants were unable to provoke death in mice. Nonetheless, mice death was independent of *katN*, so there must be other unidentified effectors that could contribute to EHEC strain EDL933 pathogenesis.

### Identification of Putative Effectors Using a Bioinformatics Approach

**Figure 2:**
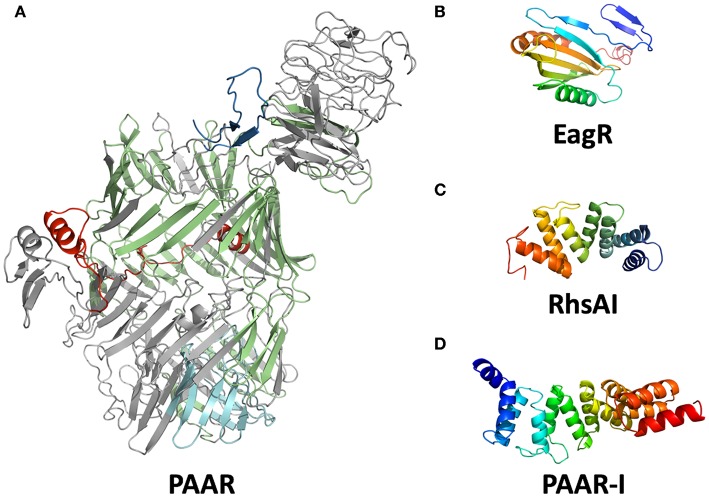
Modeled structures of T6SS associated proteins in EHEC. Structure modeling was performed using I-TASSER software. **(A)** Putative structure of PAAR (locus *z0267*). The PAAR domain is shown in red; RHS repeats (15) are shown in green; amino acid kinase is shown in cyan. The TM-score for this modeling is 0.968 with Teneurin 2 (pdb: 6fb3A). **(B)** Putative conformation of EagR (locus *z0706*). **(C)** Putative structure of RhsA-I. **(D)** The structural model of PAAR-I (locus *z0268*).

Each T6SS family has characteristic effectors according to its own functionality, and even if we could not find a single O157:H7 strain with a T6SS-1 or T6SS-3, we decided to look for effectors of any T6SS family in EHEC O157:H7 trying to determine the role of the secretion system. The commonly reported T6SS effectors in *E. coli* were not found in the genomes of EHEC O157:H7 available to date. EHEC O157:H7 lacks the effectors of the VT1-VT5 families described previously (Ma et al., 2018), as well as effectors with DUF2169, DUF4123 or DUF2345 domains. Proteins with the characteristic GXSXG or HXKXXXXD motifs were also absent (Lien and Lai, 2017). On the other hand, after identifying a PAAR domain-containing protein in the T6SS island of EHEC strain EDL933, we looked for other PAAR domain-containing proteins in EHEC strain EDL933 genome and found seven genes that encode these proteins. PAAR proteins have been found in T6SS-associated effectors in *Pseudomonas aeruginosa* and *Vibrio cholerae* (Shneider et al., 2013), in EHEC strain EDL933, they also contained RHS (Recombination Hotspot) repeats, so they were classified as Rhs family proteins. In recent years, several T6SS-effectors related to Rhs proteins have been described in UPEC and STEC strains (Poole et al., 2011; Ma et al., 2017b), as well as in other species such as *P. aeruginosa* and *S. marcescens* (Kung et al., 2012; Diniz and Coulthurst, 2015). By using the I-TASSER software, we modeled the tridimensional structure of Z0268 (PAAR) and found that this protein resembled a shell-like structure built up by β-sheets that enclose a C-terminal region with enzymatic activity (Figure 2A). The N-terminal and central region of PAAR-Rhs proteins is present in both Gram-negative and Gram-positive bacteria, and there are even homologous proteins in eukaryotes, known as teneurins. The structure of the extracellular domain of teneurins is similar to Rhs proteins, and their presence in eukaryotes was probably due to horizontal gene transfer (Jackson et al., 2018). Teneurins bind to latrophilins and mediate cell-cell adhesion (Cruz-Ortega and Boucard, 2019), while the Rhs proteins in bacteria seem to mediate intercellular competition (Koskiniemi et al., 2013).

The C-terminal region of Rhs proteins frequently share homology with several bacterial toxins and the studied Rhs effectors usually fall into the canonical categories (nucleases, lipases, and murein-hydrolases). Ma et al. (2017b) showed that the PAAR domain was required for the translocation of Rhs proteins via T6SS. The PAAR domain interacts with the C-terminal end of VgrG for its transport to the cytoplasm of the prey cell, where the Rhs C-terminal exerts its functional role (Busby et al., 2013; Shneider et al., 2013).

**Figure 3:**
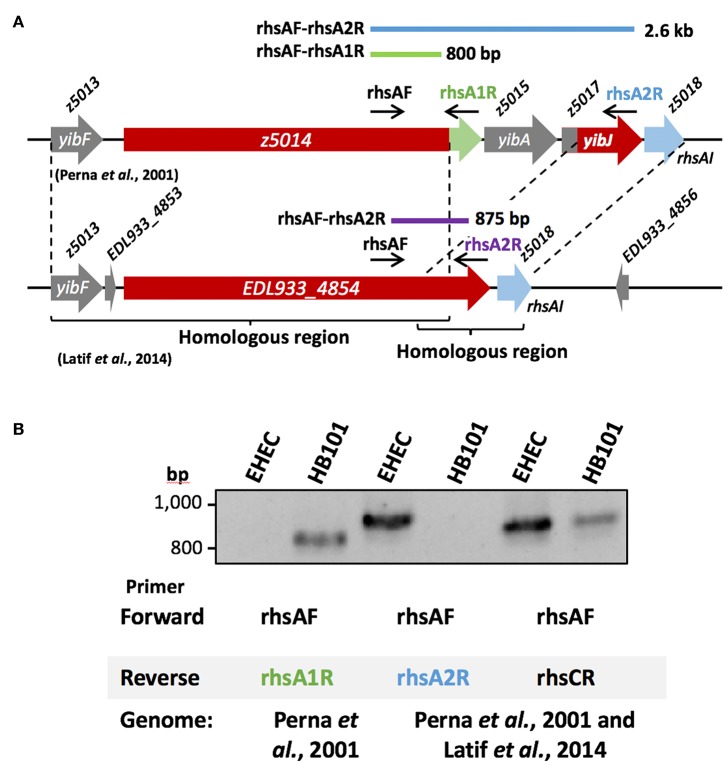
Gene amplification of rhsA in EHEC strain EDL933. **(A)** Diagram of the region between loci *z5013* and *z5018* according to Perna et al. (2001) and according to Latif et al. (2014). The *rhs* gene (red) has a different C-terminal end in each genome, harboring a DNAse domain (green) in and the sequence corresponding to locus *z5017* (red). **(B)** The 800 bp amplicon for *rhsA* gene (locus *z5014*) was not generated in EHEC strain EDL933 using the primer pair rhsAF-rhsA1R designed for the gene according to Perna et al. (2001). *E. coli* strain HB101 was used as a positive control for these primers (rhsAF-rhsA1R), which generated an 800 bp amplicon. Whereas, when the primer pair rhsAF-rhsA2R designed based on Perna et al. (2001) and Latif et al. (2014) was used, the expected 2.6 kb fragment was not obtained, but the 875 bp was, as expected from the genome from Latif et al. (2014). By using a specific primer (rhsCR) for *rhsC*, a 832 bp fragment was obtained, as expected from the genome of Latif et al. (2014), while the 2.7 kb fragment from the genome of Perna et al. (2001) was not obtained neither in EHEC EDL933 nor in *E. coli* strain HB101. A 63°C temperature was used for **(B)**. The PCR products were separated in an 1.5% agarose gel and stained using ethidium bromide.

In EHEC strain EDL933, we found several genes of the Rhs family, but these genes differ between both available genomes; 9 loci (Latif et al., 2014) vs. 11 loci (Perna et al., 2001) (Table 1). Although *z2488, z0651, z0268, z0272, z2257*, and *z0705* genes were identical in both genomes, we found several important differences. In the first genome published, for example, locus *z5014* encodes an Rhs protein with a PAAR motif that we identified as a putative DNAse domain in its C-terminal end, while in the genome published by Latif et al. (2014), this C-terminal end was not a DNAse and contained the sequence corresponding to locus *z5017* (Figures 3A,B). We also found that the protein encoded by locus *z5014* had different C-terminal regions, whereas locus *z2257* from Latif et al. (2014) genome contained regions from loci *z2256, z2257*, and *z2259* (Table 1), as there were 20 gaps and 42 mismatches between this region in both genomes. A possible reason for these discrepancies is that the genome published by Perna et al. (2001) might have assembly mistakes in the regions that encode Rhs proteins due to their size and the presence of multiple homologs in the genome. In fact, upon analysis of *rhs* genes in EHEC O157:H7 strain Sakai, we found that they were practically identical to those in EHEC strain EDL933 according to the genome published by Latif et al. (2014), both in the number of loci as in their sequences, including the C-terminal encoding region.

**Table 1:** Comparison between Rhs proteins from two available genomes in EHEC EDL933.

**Proposed name**	**Loci from Perna et al**.	**GenBank ID**	**Loci from Latif et al**.	**Genbank ID**	**Notes**
RhsA	*z5014*	AAG58737	*EDL933_4854*	AIG70995	Fused with *z5017*
RhsB	*z5488*	AAG59133	*z5488*	AIG71408	99.7% Identical
RhsC	*z0847*	AAG55021	*z0847*	AIG66972	Fused with *z0851*
RhsD	*z0651*	AAG54854	*z0651*	AIG66789	100% Identical
RhsE	*z2257*	AAG56314	*z2257*	AIG68376	*z2257*-*z2261* are fused
	*z2259*	AAG56315	*z2259*		
	*z2261*	AAG56316	*z2261*		
RhsF	*z0705*	AAG54900	*z0705*	AIG66446	100% Identical
RhsG	*z0702*	AAG54898	*z0702*	AIG66832	100% Identical
RhsH	*z0272*	AAG54539	*z0272*	AIG66830	100% Identical
PAAR	*z0268*	AAG54536	*z0268*	AIG66450	100% Identical

To test which of the two published genomes represented better the *rhs* genes in EHEC strain EDL933, we decided to amplify a region of an *rhs* gene present in EHEC EDL933 that should correspond to the same sequence in the different genomes reported; in Perna et al. (2001), it would be the locus *z5014* and in Latif et al. (2014) the locus *EDL933_4854*. Locus *z5014* from the genome published by Perna et al. (2001) contains a 3′ end that does not correspond to any other sequence from the genome published by Latif et al. (2014), while in this latter genome the locus *z5017* is fused to the 3′region of *z5014*, here named *EDL933_4854* (Figure 3A). Thus, we designed a forward primer (rhsAF) that could serve for both genomes, as well as a reverse primer (rhsA1R) specific for the 3′region of *z5014* in Perna et al. (2001) that would produce an 800 bp amplicon. We also designed another reverse primer (rhsA2R) that aligned with locus *z5017* from Perna et al. (2001) and locus *EDL933_4854* from Latif et al. (2014), which would produce a 2.6 kb long amplicon and an 875 bp long amplicon, respectively (Figure 3A). The reverse primer designed for the genome published by Perna et al. (2001) (rhsA1R) did not amplify the expected DNA fragment (800 bp) in EHEC EDL933 (Figure 3B), while the reverse primer for the genome published by Latif et al. (2014) (rhsA2R), amplified the 875 bp fragment from EHEC strain EDL933. To test if the reverse primer designed for the genome published by Perna et al. (2001) was reliable, we used *E. coli* strain HB101 as a positive control, since this strain has an *rhs* gene identical to the locus *z5014* reported by Perna et al. (2001). The amplification of an 800 bp fragment only in strain HB101 using rhsA1R primer (Figure 3B) suggested that EHEC EDL933 does not harbor the sequence reported by Perna et al. (2001). Using the same strategy (see Figure 3A), we analyzed the *rhsC* gene region, another chromosomal region with differences between both genomes. The region of the *rhsC* gene was amplified using the rhsAF primer and a specific primer (rhsCR) that would give a 2.7 kb fragment for the genome of Perna et al. (2001) and a 832 bp fragment for the genome of Latif et al. (2014). Similar to *rhsA*, a fragment of 832 bp was obtained both in EHEC ELD933 as in *E. coli* strain HB101, while the 2.6 kb fragment was not obtained in any strains. In conclusion, the genome published by Latif et al. (2014) better represents the *rhs* genes in EHEC strain EDL933, so we recommend using this genome in posterior analyses. In this genome, we found 9 genes of the *rhs* family, of which 7 are long (>4 kb) and have a PAAR domain, while the two remaining are relatively small (<1 kb) and do not have a PAAR domain. Because the locus *ELD933_4854* was similar to the *rhsA* gene from *E. coli* strain K-12, we named this gene as *rhsA*, named the other loci as *rhsB-H* according to their sequence similarity with genes in *E. coli* K-12, and named *paar* the *rhs* gene that is encoded inside the T6SS island to differentiate it from the others.

Once we determined which genome was better for the study of the Rhs family proteins, we wanted to elucidate the possible role of each protein, so we analyzed the C-terminal end of each Rhs looking for functional domains, and for effectors structural homology using I-TASSER software. We first analyzed the RhsA protein (locus *EDL933_4854*) and did not find any known motif, but after modeling its structure with I-TASSER we found that the C-terminal region had structural similarity with the toxin-A of *Clostridium difficile*, a toxin that inactivates GTP-binding proteins such as Rho, Rac, and Cdc42 (Voth et al., 2019) (pdb: 3HO6, TM-score: 0.73). The C-terminal end of RhsB (*z5488*) was structurally similar with the human proteasome 26S lid, which recognizes ubiquitinated proteins (Schweitzer et al., 2016) (pdb: 5l4kV, TM-score: 0.694), though we found a DUF4329 motif (PF05593, *e-value*: 5x10^−13^) that according to Ma et al. (2017b) is an RNase domain. The C-terminal end of RhsC (*z5014*) had structural similarity with VipE protein of *Legionella pneumophila*, a protein that inhibits the vesicular traffic in yeast (Shohdy et al., 2005) (pdb: 4qn8A, TM-score: 0.574). The C-terminal region of RhsD (*z0651*) was structurally homologous with the human serum amyloid A1 protein (Lu et al., 2014) (pdb: 4ip8A, TM-score: 0.623), which participates in the inflammatory response, chemotaxis and opsonization upon binding with several integrins and G protein-coupled receptors (GPCRs) (Niemi et al., 2011). The C-terminal end of RhsE (*z2257*) had structural homology with the RsaA protein of *Caulobacter crescentus* (Bharat et al., 2017) (pdb: 5n8pA, TM-score: 0.71), as well as the adhesin PfbA of *Streptococcus pneumoniae* (Suits and Boraston, 2013) (pdb: 3zpp, TM-score: 0.647). Both RsaA and PfbA are present in the S-layer of bacteria and could serve as adhesins. Finally, the C-terminal end of PAAR (*z0268*) presented homology with the cysteine-protease domain of the RtxA toxin of *V. cholerae* (Prochazkova et al., 2009) (pdb: 3fzyA, TM-score: 0.681), a toxin that crosslinks actin and actively participates during the pathogenesis of *V. cholerae*. Nonetheless, the C-terminal end did not have a RTX motif nor the actin cross-linking domain normally found in the RtxA toxin (Boardman et al., 2007) (Table 2).

**Table 2:** Putative function of Rhs effectors in EHEC strain EDL933.

**Locus**	**Domain homolog**	**TM-score**	**Homolog function**
EDL933_4854 (rhsA)	Toxin A from *C. difficile*	0.73	Small-GTPase inhibition (like Rho and Rac)
z5488 (rhsB)	Proteasome lid 26S	0.694	Protein ubiquitination
z5014 (rhsC)	VipE	0.574	Vesicular-traffic inhibition
z0651 (rhsD)	human serum amyloid A1 protein	0.623	Chemotaxis, inflammatory response
z2257 (rhsE)	RsaA from *Caulobacter crescentus*	0.71	S-layer Adhesin
z0268 (paar)	Toxin RtxA from *V. cholerae*	0.681	Cysteine protease, actin-crosslinking
z0705 (rhsF)	Capsid protein from betanodavirus	0.863	Capsid protein

RhsF protein (*z0705*) did not harbor any apparent motif, but the structural modeling showed that the C-terminal end resembled the structure of capsid proteins present in viruses such as betanodavirus (pdb: 3jbmA, TM-score: 0.863) or Orsay-like virus (pdb: 4nwvA, TM-score: 0.825). Just downstream of the *rhsF* gene, we found another *rhs* gene (*rhsG*, locus *z0702*). RhsG protein had a metallopeptidase-4 domain (PF15640, *e-value*: 1 × 10^−32^), which has already been described in a similar STEC strain (Ma et al., 2017b), but lacked the PAAR domain in EHEC strain EDL933 and it seems to be interrupted (this region is identical in both published genomes). The presence of a C-terminal region similar to capsid proteins has not been reported previously, and upon inspecting the 3′ region of the *rhsF* gene and the linker sequence between *rhsF* and *rhsG*, we found that both sequences shared similarity with Pithovirus LCDPAC02. This suggests that the *rhsG* gene was originally fused with *rhsF*, but a viral sequence was inserted in this region and separated the gene. It is unclear if the C-terminal of the RhsF protein has a function related to interbacterial competition or pathogenesis. To corroborate the predicted functions of the Rhs proteins as T6SS effectors, a translocation assay is needed, and this becomes complicated due to the current difficulty to activate the T6SS *in vitro*. As there are several methodologies to achieve expression of the T6SS *in vitro* (Gueguen and Cascales, 2013; Miyata et al., 2013; Wan et al., 2017), co-expression of the Rhs proteins and the T6SS components needs to be achieved to properly investigate their function. Heterologous expression can help to study the effector function, and murine infection models with isogenic mutants for these genes could help to establish the importance of the Rhs proteins during intestine colonization and pathogenesis.

**Figure 4:**
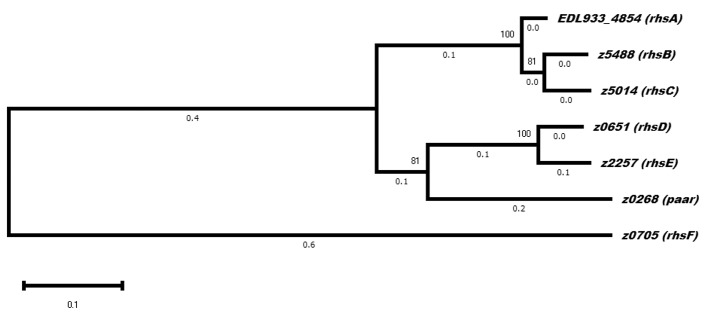
Evolutionary analysis of *rhs* genes in EHEC strain EDL933. The phylogenetic tree shows three distinct clades, of which the one containing *rhsF* is the one that diverged first. Two of the genes encoded in a *vgrG* island are in the same clade (*rhsE* and *paar*), while *rhsA*. *rhsB* and *rhsC* did not belong to a neighborhood containing other T6SS genes. The evolutionary history was inferred by using the Maximum Likelihood method and Tamura-Nei model. The tree with the highest log likelihood (-23369.81) is shown. The percentage of trees in which the associated taxa clustered together is shown next to the branches. Evolutionary analyses were conducted in MEGA X (Kumar et al., 2018).

The genes *rhsE, rhsF*, and *paar* were located downstream of a distinct *vgrG* gene (Figure 1), suggesting that these Rhs proteins are translocated by a its specific VgrG protein. On the other hand, *rhsA, rhsB, rhsC*, and *rhsD* were orphan genes that did not belong to a *vgrG* island, so presumably, they can be translocated by any VgrG protein. If each VgrG protein translocates specific Rhs proteins, then it would be logical to think that the *rhs* genes are divided in three categories according to the number of *vgrG* genes in EHEC EDL933. Using phylogenetic analysis, we found that *rhs* genes were indeed divided into three clades (Figure 4). The first clade grouped *rhsA, rhsB*, and *rhsC* genes being the last two more closely related. The second clade contained *rhsD, rhsE*, and *paar* genes, being the latter one more dissimilar of them. Finally, *rhsF* gene was clearly different from the others, being the only member of its clade. While *rhsF* is adjacent to *vgrG1*, it would seem that VgrG1 translocates RhsF, however, both *rhsE* and *paar* are in the same clade and are adjacent to *vgrG2* and *vgrG3*. Besides, the first clade containing *rhsA, rhsB*, and *rhsC* did not include any *rhs* gene encoded around a *vgrG* island, so it is unclear which VgrG protein, if any, translocates these proteins. Ma et al. (2017b) claimed that, in STEC, the *rhs* genes encoded in the vicinity of the EagR chaperone are phylogenetically distinct from the orphan genes (*rhs* genes not encoded in an *eagR* island), which is consistent in EHEC EDL933, as *eagR* was directly upstream of the *rhsF* gene, the less conserved gene in EHEC EDL933. When we modeled the structure of EagR (locus *z0706*) (Figure 2B), we found that the protein resembles PA0094 (TM-score: 0.888) from *P. aeruginosa*, whose crystal structure was obtained by Osipiuk et al. (unpublished, pdb: 1TU1). PA0094 acts as a chaperone for the Tse6 (PA0093), a PAAR-domain containing protein that also has a Ntox46 domain with nuclease activity. The high structural homology between PA0094 and EagR suggests that the EagR protein also functions as a chaperone for RhsF in EHEC EDL933. Nevertheless, because of the absence of a known motif in RhsF C-terminus, more studies are needed to determine if this island and its encoded proteins are indeed functional.

We have found that *rhs* genes are widely distributed among bacteria through data base analyses. By searching all EHEC O157:H7 genomes in GenBank, we found that there are some strains having up to 11 of these genes. Other studies have already classified PAAR-Rhs proteins as T6SS effectors, being mostly nucleases or peptidases (Koskiniemi et al., 2013; Ma et al., 2017b). The RNAse motif present in Z5014 from the genome published by Perna et al. (2001) (PF15606) is not present in other Rhs proteins from O157:H7 strains, suggesting that this motif is not present in EHEC O157:H7. However, in EHEC O157:H7 strains there are 9 PAAR-Rhs proteins with a DUF4329 that has a RNase activity, including RhsB from EDL933 as showed above (*e* ≤ 0.01). Interestingly, 7 PAAR-Rhs proteins in EHEC O157:H7 strains contain a serum amyloid A protein motif in their C-terminal region (PF00277; *e* ≤ 0.01). As we discussed earlier, this motif could play a role in the immunological response but experimental evidences are required. Though we found 26 Rhs proteins with metallopeptidase-4 domain in EHEC O157:H7 strains, none had a PAAR domain that would identify them as T6SS effectors. Finally, 11 PAAR-Rhs proteins contained an amino acid kinase motif (AAK, cd04259) in their C-terminal, which is commonly found in proteins related to lysine and aspartate metabolism. Proteins with this activity could phosphorylate prey proteins and change their behavior, thus contributing to the bacterium survival and/or pathogenesis. However, EHEC O157:H7 strain EDL933 does not possess this interesting motif.

### Immunity/Effector Rhs Pairs in EHEC Strain EDL933

Antibacterial effectors translocated via T6SS are usually encoded by a bicistronic operon containing genes for an effector and its respective immunity protein, whose function is to inhibit the activity of this effector. These immunity proteins are useful to avoid autointoxication and to guard off attacks of sister cells. When we analyzed the region downstream of the *rhs* genes, we found that all of them contain a relatively small ORF (129-609 nucleotides) that could act as the immunity gene for each Rhs. Just as the C-terminal ends are highly divergent, these related immunity proteins are also poorly conserved, suggesting that an immunity protein is only able to inhibit the action of its related effector, as demonstrated for *Dickeya dadantii* strain 3937 (Koskiniemi et al., 2013). To know if the genes downstream *rhs* genes encoded immunity proteins, we decided to look for protein-protein interaction motifs as well as structural modeling of the putative proteins to search structural homologs in the same fashion as we did for the C-terminal end of the Rhs proteins.

Locus *z5018* would encode for the putative immunity protein of RhsA, a 132 amino acid protein that we named RhsAI. In this protein, we found tetratricopeptide repeats (TPR; *e-value*: 1 × 10^−5^) and Sel-1-like repeats (SLR; *e-value*: 4 × 10^−5^). TPR and SLR motifs belong to the Sel-1 superfamily, whose members usually act as molecular adaptors between proteins both in prokaryotes and eukaryotes (Mittl and Schneider-Brachert, 2007). Recently, the crystal structure of two immunity proteins for T6SS effectors was obtained from *P. aeruginosa* (PA5088 and PA5087), and these structures harbor SLR repeats that directly interact and inhibit phospholipase D effectors (Yang et al., 2016). Nonetheless, PA5087 and PA5088 SLR motifs only possessed 35.06 and 28.99% identity with RhsAI, respectively. Through modeling RhsAI structure using I-TASSER (Figure 2C), we found that it shares structural homology with the protein C5321 from Uropathogenic *E. coli* (UPEC) strain CFT071 (TM-score: 0.899, pdb: 4BWR). Urosev et al. (2013) described C5321 as a potential model for vaccine-development against ExPEC, but did not provide any more information about the protein function in UPEC. We did not find any known T6SS effector in the vicinity of *c5321* in UPEC, nor any *rhs* gene, so it seems unlikely that this protein acts as an immunity protein in UPEC. Nonetheless, it could be possible that *c5321* is an orphan immunity gene and that the presence of this gene might protect UPEC from RhsA of EHEC, or other T6SS effectors in gram-negative bacteria. RhsAI also possesses structural homology with the protein LpnE from *Legionella pneumophila* (TM-score: 0.899, pdb: 6dehA). LpnE interacts with the Oculocerebrorenal syndrome of Lowe (OCRL) protein in eukaryotic cells (Voth et al., 2019), which is a Inositol-polyphosphate 5-phosphatase. This similarity was interesting because the PA5088 immunity protein (which has structural homology with RhsAI) also interacts with a phosphatase effector, suggesting that RhsA might have a phosphatase activity, and phosphatases have been implicated in antibacterial activity, pathogenesis, or even both (Jiang et al., 2014). Finally, RhsAI had also structural homology with the protein Suppressor of lin-12-like protein 1 (SEL1L), a murine protein that can oligomerize, something that is also necessary for some immunity proteins to inhibit T6SS-associated effectors (Yang et al., 2018).

The putative immunity protein for RhsB only contained 57 amino acids (locus *z5489*), and upon sequence analysis, we found two interesting motifs. The first one is from the family PF07114 including the transmembrane protein 126 (TMEM126A/B, *e-value*: 0.022), which suggests its possible location in the cell, although the *e-value* is high, it is on the edge. In humans, TMEM126B participates in the construction of the mitochondrial respiratory complex I (Andrews et al., 2013). The membrane location should be congruent with the predicted function of RhsB as ubiquitin-receptor, as we predicted above using structural modeling, and the TMEM family have a variety of functions in eukaryotes, participating in phospholipid scrambling, ion transport and regulation of other membrane proteins (Pedemonte and Galietta, 2014). On the other hand, RhsBI also contained a dsRNA-binding domain found in the Dead-end protein homolog-1 (DND1) protein (*e-value*: 0.087). Both motifs have a high *e-value*, meaning that RhsB shares poor homology with those proteins, so the function of the RhsBI protein remains unclear until experimental evidence. However, it is worth to mention that DND1 is a RNA-binding protein, and it is known to protect eukaryotic mRNA from miRNAs, so the RhsBI protein could have a protective effect from mRNA degradation from the RNase motif DUF4329 present in RhsB, as we discussed earlier.

For RhsCI (locus *z0853*) and RhsDI (locus *z0654*) there was a TPR motif and a TIGR03373 motif, respectively, but both had high *e-values* (0.21 and 0.35), suggesting that there is no sequence similarity between these proteins and the consensus sequence. STRING analyses suggest that the *rhsC-D* genes are often in the same neighborhood as their cognate immunity genes, but there is no evidence of co-expression or interaction for any Rhs protein with their associated immunity protein.

Locus *EDL933_2188* would encode the immunity protein of RhsE, and the protein encoded was of 61 amino acids that bear a lipoprotein signal peptide according to the software SignalP (Juncker et al., 2003), indicating that the immunity protein is probably anchored to the bacterial internal membrane. As we mentioned earlier, RhsE protein possessed structural homology with RsaA protein, present in the S-layer. S-layer located proteins (SLPs) are involved in several activities, such as cell-wall biogenesis, cell division, and swimming. Besides, SLPs have been involved in pathogenesis, altering the immune response of the host and promoting bacterial adhesion to host cells (Fagan and Fairweather, 2014). This signal peptide might lead to the translocation of RhsEI to the periplasm, suggesting that RhsEI binds to RhsE in the outer membrane, where RhsE would normally act. Dong et al. (2013) have shown that Tai3, the immunity protein for the amidase effector Tae3 in *Ralstonia picketti*, also harbors a signal sequence. In the S-layer, the C-terminal end of RhsE could damage the plasma membrane or the cell-wall of other bacteria, and the immunity protein would be on the membrane to avoid cell death provoked by RhsE in sister cells. Additionally, RhsEI possessed another motif (PF16855) present in small proteins of the viral external capsid (*e-value:* 0.072), though the high *e-value* indicates poor homology with the consensus sequence of PF16855.

Finally, the candidate for the PAAR-I protein (locus *z0269*) was 203 amino acid long, larger than other putative immunity proteins. We identified 7 SLR/TPR motifs, as previously mentioned, are motifs found in immunity proteins. Upon analysis of the putative tridimensional structure of PAARI by I-TASSER, we found that the immunity protein resembled the *Helicobacter* cysteine-rich protein C (TM-score: 0.928, pdb: 1OUV) (Figure 2D). Although the exact role of this protein is still unknown, it seems to participate during cell-wall biogenesis. Besides, patients infected with *H. pylori* usually bear high antibody titers against this protein. Lüthy et al. (2004) demonstrated that the structural conformation of the *Helicobacter* cysteine-rich protein C resembles the Hsp60/Hsp70, which provides more evidence of the possible chaperone activity of PAARI. Finally, the presence of loci *z0268* and *z0267* is specific to a specific lineage of O157:H7 that includes EHEC EDL933 and EHEC Sakai, as previously reported (Zhang et al., 2007).

Small Rhs proteins were also part of bicistronic operons, and we named them RhsH (*z0272*) and RhsG (*z0702*). Both effectors were part of an island that also contained another *rhs* gene. RhsG seems to be the former C-terminal region of RhsF that was probably divided when a viral sequence was inserted, as we mentioned above. Since these proteins were 444 and 586 amino acids in length, compared to the >1,400 amino acids of other Rhs proteins, their genes were probably part of a larger former gene. The putative immunity proteins for RhsG and RhsH were also small (69 and 56 amino acids respectively) and did not have a clear structural homolog. The absence of the PAAR motif in both proteins suggests that these proteins are not secreted via T6SS, and the small size and poor structural homology of the putative immunity proteins suggest that their genes are actually pseudogenes.

The immunity proteins protect the bacteria from sister cells attacks when the effectors modulate antibacterial competition. Interestingly, Wan et al. (2017) have shown that EHECΔ*hns* does not have a bactericidal activity, suggesting that the Rhs proteins do not have a toxic effect on other bacteria, although *rhs* gene expression was not measured. As we show here, several effector-immunity proteins seem to be related to the cell membrane and cell-wall biogenesis, so more studies are necessary to elucidate the function of the T6SS both in bacterial competition and during pathogenesis. It is also possible that the putative immunity proteins described have a different function than predicted, maybe as chaperones like EagR.

## Conclusions

The two existing genome sequences exhibit critical differences among the Rhs proteins, and as demonstrated experimentally and bioinformatically we suggest using the genome published by Latif et al. (2014) to study the Rhs proteins in EHEC strain EDL933. Upon analysis of the core components of the T6SS island, we found that the TssA gene seems to be split in two, but these two proteins should still be able to perform their function, as the functionality of the T6SS has been demonstrated by Wan et al. (2017). Although EHEC strain EDL933 lacks the commonly found T6SS effectors in *E. coli*, many *rhs* genes appear to serve as T6SS effectors. Using a structural homology approach, we postulate that RhsA may interact with small GTPases such as Rho and Rac; RhsB could interfere in the protein ubiquitination; RhsC might participate in the vesicular traffic; RhsD could auto-aggregate; RhsE might act as an S-layer adhesin; and PAAR could act as a cysteine protease. Finally, RhsF would not have any function due to a viral DNA sequence inserted on the 3′ end of the gene, which separated the former metallopeptidase into RhsF and RhsG. Additionally, we did not find direct *in silico* information supporting EHEC strain ELD933 is involved in bacterial competition, as suggested by Wan et al. (2017). The role of Rhs proteins during pathogenesis or bacterial competition requires experimental support. The exact role of T6SS and its effectors in EHEC and other STECs might provide new strategies to fight diseases caused by those pathogens.

## Data Availability Statement

Publicly available datasets were analyzed in this study. This data can be found here: AE005174.2, CP008957.1.

## Author Contributions

JV-L and FN-G participated in the design of the study, data analysis and writing of the manuscript. JV-L carried out the PCR experiments. Both authors read and approved the final manuscript.

## Conflict of Interest

The authors declare that the research was conducted in the absence of any commercial or financial relationships that could be construed as a potential conflict of interest.
